# Single-cell RNA sequencing revealed PPARG promoted osteosarcoma progression: based on osteoclast proliferation

**DOI:** 10.3389/fimmu.2024.1506225

**Published:** 2025-01-28

**Authors:** Lei Sun, Jinhao Zhang, Zhikai Xiahou, Zhenzhen Zhao, Yanchen Liang

**Affiliations:** ^1^ Shandong University of Traditional Chinese Medicine, Jinan, Shandong, China; ^2^ School of Stomatology, Southwest Medical University, Luzhou, Sichuan, China; ^3^ China Institute of Sport and Health Science, Beijing Sport University, Beijing, China; ^4^ Department of Orthopedics, Affiliated Hospital of Shandong University of Traditional Chinese Medicine, Jinan, Shandong, China

**Keywords:** single-cell sequencing, osteoclasts, osteosarcoma, cell communication, transcription factors, novel targets, experiment validation

## Abstract

**Background:**

Osteosarcoma (OS) is one of the most common primary malignant bone tumors, primarily originating from mesenchymal tissue. It is notorious for its high invasiveness, high disability rate, high mortality rate, and poor prognosis. In most primary and metastatic malignant tumors, bone destruction can promote cancer progression, which is closely related to osteoclast activation and the imbalance between osteoblasts and osteoclasts. A large number of studies confirmed that osteoclasts are an important part of OS, which play an active role in destroying bone homeostasis and promoting the progress of OS. Therefore, we conducted a detailed study of osteoclasts at the single cell level, aiming to find new OS therapeutic targets to prevent tumor progression and local spread.

**Methods:**

We analyzed the single-cell sequencing data of OS patients and usedMonocle2, Cytotrace, and Slingshot software to analyze the pseudo-sequential trajectory during OS progression. CellChat was used to reveal the communication between cells. PySCENIC was used to identify active transcription factors in osteoclasts. Finally, we further demonstrated the results by RT-qPCR analysis, CCK-8 assay, wound healing assay, Transwell assay, etc.

**Results:**

Through the analysis of single-cell sequencing data in OS, we identified a highly specific subgroup, C2MKI67+ Osteoclast. The key signaling pathway APP and the top 1 transcription factor PPARG in this subgroup played essential roles in osteoclast proliferation and differentiation. Given the pivotal role of osteoclasts in OS progression, we speculated that these signaling pathways and transcription factors could emerge as novel therapeutic targets, offering innovative strategies for OS treatment.

**Conclusion:**

This study enhanced our understanding of OS and osteoclasts through scRNA-seq. Furthermore, we discovered that PPARG amplifies osteoclast activation and proliferation, resulting in excessive bone resorption and degradation of the bone matrix, thereby creating a favorable environment for tumor cell proliferation and growth. By innovatively targeting PPARG, it affected osteoclast proliferation and thus affected tumor progression; this work offered new insights and directions for the clinical treatment of OS patients.

## Introduction

Osteosarcoma (OS) (1) is one of the most prevalent primary malignant bone tumors, originating predominantly from mesenchymal tissue. It is characterized by high aggressiveness, high disability rates, high mortality rates, and a poor prognosis, with pulmonary metastases potentially emerging within months (2, 3). OS was one of the common pediatric cancers, which occured in children and adolescents (4–8). The typical sites of OS onset are the long bones, most commonly affecting the distal femur, proximal tibia, and proximal humerus; they account for about 85% of all limb OS (9). Patients commonly present with symptoms such as pain, palpable masses, and other systemic manifestations (7). OS patients are classified into two categories: localized and metastatic. Localized OS accounts for about 80% of cases, with a five-year overall survival rate below 70% and an amputation rate between 10% and 20% (10, 11). Furthermore, about 70% of metastatic OS patients experience pulmonary metastases, with a five-year survival rate of less than 30% (11). Among all OS cases, the classical subtype constitutes approximately 70%, further divided into osteoblastic, chondroblastic, and fibroblastic variants. This study focuses on primary osteoblastic and primary chondroblastic classical OS, employing single-cell transcriptomics for in-depth analysis (12). Currently, surgery remains the cornerstone of OS treatment, with chemotherapy and radiotherapy serving as adjuvant therapies. A multimodal approach involving neoadjuvant chemotherapy (pre-surgery), surgical resection, and adjuvant chemotherapy (post-surgery) is commonly employed to achieve optimal treatment outcomes (13, 14). Advances in gene testing, targeted therapy, immunotherapy, CAR-T therapy, and stereotactic radiotherapy have significantly extended survival times and improved the five-year survival rates of OS patients. Concurrently, the development of novel therapeutic agents for OS has progressed markedly. Commonly used drugs include doxorubicin, cisplatin, methotrexate, cyclophosphamide, epirubicin, carboplatin, and apatinib (15).

Targeted therapy, celebrated for its efficiency, low toxicity, and convenience, has rapidly evolved. A large number of tyrosine kinase inhibitors are under investigation for OS, with notable examples including anti-angiogenic agents like pazopanib, sorafenib and apatinib, and others. Despite these advancements, many patients still face challenges in obtaining effective diagnosis and treatment. OS is marked by strong chemoresistance, high recurrence, and a proclivity for metastasis, leaving survival rates suboptimal. Thus, enhancing early diagnosis, suppressing recurrence and metastasis, and improving prognosis remain urgent and formidable challenges addition, we also use a multimodal approach involving neoadjuvant chemotherapy, surgery, and adjuvant chemotherapy to achieve the purpose of treating OS. Although multimodal treatment significantly improved the 5-year survival rate of OS patients, a substantial number still experienced recurrence and metastasis, and the survival rate remained suboptimal (16, 17).

The tumor microenvironment (TME) (18–21), composed of tumor cells, tumor-associated fibroblasts, immune cells, endothelial cells, various other cell types, and non-cellular components, creates a complex and dynamic ecosystem (22). In most metastatic malignancies, disruption of bone matrix integrity is closely linked to cancer progression (20, 23). Overactivation of osteoclasts and suppression of osteoblasts are pivotal factors driving bone metastases (24). In OS, osteoclasts play a crucial role; their hyperactivity can lead to degenerative bone diseases such as arthritis and osteoporosis (25–27). Research indicates that osteoclasts promote bone resorption through their osteolytic activity, disrupting bone homeostasis and contributing to bone metastases in malignancies (28, 29). Osteoclasts interact with various cell types in the OS TME, particularly OS cells and osteoblasts, with key signaling pathways, such as the Wnt/β-catenin pathway (30) and the RANK-RANKL pathway (31), regulating their activity and involvement in bone metastases. Additionally, osteoclasts secrete cytokines that further accelerate OS progression (32, 33). While some studies suggest that osteoclasts are potential therapeutic targets for OS, research on inhibiting their overactivation remains insufficient, presenting new therapeutic opportunities for OS treatment (34, 35).

Single-cell sequencing (scRNA-seq) (36–41), a high-throughput method for analyzing gene expression at the single-cell level, has been instrumental in deciphering the cellular composition of the TME in OS patients. This study utilizes scRNA-seq to explore the heterogeneity of osteoclasts, identifying key subpopulations and specific targets. Experimental validation confirms these targets’ essential roles in osteoclast proliferation, activation, and migration. These findings offer novel insights for clinical treatment of OS, aiding in the optimization of diagnostic tools and the development of more precise therapeutic strategies.

## Methods

### OS data source

scRNA-seq data was obtained from the Gene Expression Omnibus (GEO) database (https://www.ncbi.nlm.nih.gov/geo/), accession number GSE152048. Since the data comes from a public database, ethical approval was not required.

### ScRNA-seq data processing and cell type identification

The Seurat package (version 4.3.0) was used to analyze OS data (37). First, the DoubletFinder program (version 2.0.3) was employed to filter out poor-quality cells, including doublets and multiplets, from the OS samples (42–44). The criteria were set as follows: 300 ≤ nFeature_RNA ≤ 7500, 500 ≤ nCount_RNA ≤ 50000, mitochondrial gene expression ≤ 25%, erythrocyte gene expression ≤ 5%, to select qualified cells. The data was normalized by the “NormalizeData”function. Next, we identified 2000 highly variable genes (HVGs) (45–47). The “scaleData” function standardized the data. Principal component analysis (PCA) (48–51) was performed on these highly variable genes using the “RunPCA” function, selecting the top 30 PCs for dimensionality reduction clustering, and harmony (version 0.1.0) was used to address batch effects. UMAP was used for dimensionality reduction clustering analysis, and the results were displayed in a two-dimensional space (52–54). For clustering the reduced data, the FindNeighbors and FindClusters functions from the Seurat package were used (55, 56). In addition, the “FindAllMarkers” function, single-cell public databases (CellMarker), and published articles were utilized to find corresponding single-cell annotation reference datasets, marker genes, and differentially expressed genes (DEGs) to improve annotation accuracy (57).

### Gene Ontology, Kyoto Encyclopedia of Genes and Genomes, Gene Set Enrichment Analysis

GO (Gene Ontology) and KEGG (Kyoto Encyclopedia of Genes and Genomes) (58–61) were commonly used databases. GO’s functional datasets (62, 63) were divided into three subclasses: Biological Process (BP), Molecular Function (MF), and Cellular Component (CC). The fixed thresholds were set as |log2FoldChange|> 1 and FDR <0.05 to screen for differentially expressed genes, followed by pathway enrichment analysis. Gene set enrichment analysis (GSEA) (64–66) sorted the predefined gene set according to the degree of differential expression in the two types of samples and evaluated the enrichment of the gene set in the ranking list, with P-values <0.05 indicating significant pathways and the normalized enrichment score (NES) ranking pathways from high to low.

### Pseudotemporal ordering of OS

The Monocle2 software (67) toolkit (version 2.22.0) was used to construct single-cell pseudotime trajectories, analyzing cellular changes during OS progression. DDRTree technology was employed for dimensionality reduction, followed by cell sorting and assigning each cell a pseudotime value. Reduced-dimension cells were displayed in a two-dimensional space, forming a tree structure, with cells at the root considered the initial state. Cells were colored based on pseudotime values for visualization. CytoTRACE evaluated stemness and differentiation potential in Osteoclasts in OS single-cell data. Slingshot(version 2.6.0) observed the cell trajectory during differentiation, which was used to infer the dynamic expression level of cells in different differentiation trajectories. The getLineages and getCurves functions constructed a minimum spanning tree (MST) based on cell clusters, determining the overall lineage structure and depicting gene expression changes over the differentiation trajectory. The results were visualized and evaluated.

### Cell-cell communication

To study intercellular communication in OS, the “CellChat” package (version 1.6.1) (68)was used, primarily exploring cell interactions, ligand-receptor pairs, and signaling pathways between osteoclasts and other cell types (67).

### Transcription factor analysis

SCENIC, based on co-expression and motif analysis, revealed gene regulatory network reconstruction and transcription factor activity. This study used the pySCENIC (version 0.10.0) package in Python (version 3.7). AUCell was mainly used to evaluate the activity of regulon in each cell.

### Cell culture and transfection

Cell culture Osteoclast cell lines were collected from American Type Culture Collection (ATCC). The cell line was cultured in human osteoclast complete medium containing 10% fetal bovine serum (FBS), 1% streptomycin, and penicillin (Gibco BRL, USA). The standard incubation conditions were 37°C, 5% CO2, and 95% humidity. In this study, PPARG knockdown was achieved using small interfering RNA (siRNA) constructs. The cells were seeded in a 6-well plate at a confluence of 50% and transfected with two small interfering RNAs that knocked down PPARG (Si-PPARG-1 and Si-PPARG-2) and a negative control (Si-NC). The steps described were performed according to Lipofectamine 3000RNAiMAX (Invitrogen, USA).

### RT-qPCR analysis

RT-qPCR was a molecular biology technique used for detecting and quantitatively analyzing the expression levels of specific genes (69). We extracted total RNA from cell lines using TRIzol reagent and subsequently reverse transcribed the mRNA into cDNA using the PrimeScript™ RT reagent kit (Vazyme, R232-01). For real-time quantitative PCR (RT-qPCR), we used the SYBR Green Kit (TaKaRa Biotechnology, Dalian, China), with GAPDH as the internal control. The specific primer sequences are listed in **Supplementary Figure 1**.

### Cell-counting kit-8 assay

The cell viability of transfected osteoclasts was assessed using the CCK-8 assay. Cell suspensions were seeded into 96-well plates (Corning, USA, 3599) at a density of 5×10³ cells per well and cultured for 24 hours. Cells were then treated with CCK-8 reagent (A311-01, Vazyme) and incubated in a dark environment at 37°C for 2 hours. On days 1, 2, 3, and 4, the absorbance at 450 nm was measured using a microplate reader. The average OD values were calculated and represented as a line graph.

### Wound healing assay

Also known as the scratch assay, the wound healing assay is commonly used to study cell migration ability and cell-cell interactions. Transfected osteoclasts were placed in a 6-well plate (Corning, USA, 3516) and cultured in an incubator until the cell density reached approximately 95%. A sterile 200 µL pipette tip was then used to create a straight-line scratch on the cell monolayer. We washed away cell debris and floating cells with PBS (phosphate-buffered saline) to avoid interference with subsequent observations. The cells were then transferred to serum-free cell culture medium and cultured. We captured images at the same location at 0 hours and 48 hours, and the change in scratch width was measured using image analysis software (ImageJ).

### Transwell assay

The Transwell assay was a commonly used *in vitro* method to study cell migration and invasion, focusing on cell migration ability and invasion ability. Transwell chambers with or without Matrigel matrix (BD Biosciences, USA). The upper chamber (using serum-free medium), the lower chamber (using complete medium), we inoculated 1 × 104 cells in the upper chamber and cultured for 48 hours. The cells were then fixed with 4% paraformaldehyde (PFA) and stained with 0.1% crystal violet (Solarbio, China). Cell counting was performed under an optical microscope, and the migrated cells were photographed and quantified.

### Statistical analysis

We used R software (version 4.3.0) and Python software (version 4.2.0). In addition, the Wilcoxon paired nonparametric test and Pearson correlation coefficient were used in the study (70). Statistically significant data with P-values <0.05 were considered, including * p < 0.05, ** p < 0.01, *** p < 0.001, **** p < 0.0001, ns indicated no statistical difference.

## Results

### Identification of cell types and heterogeneity in OS

**Figure 1:**
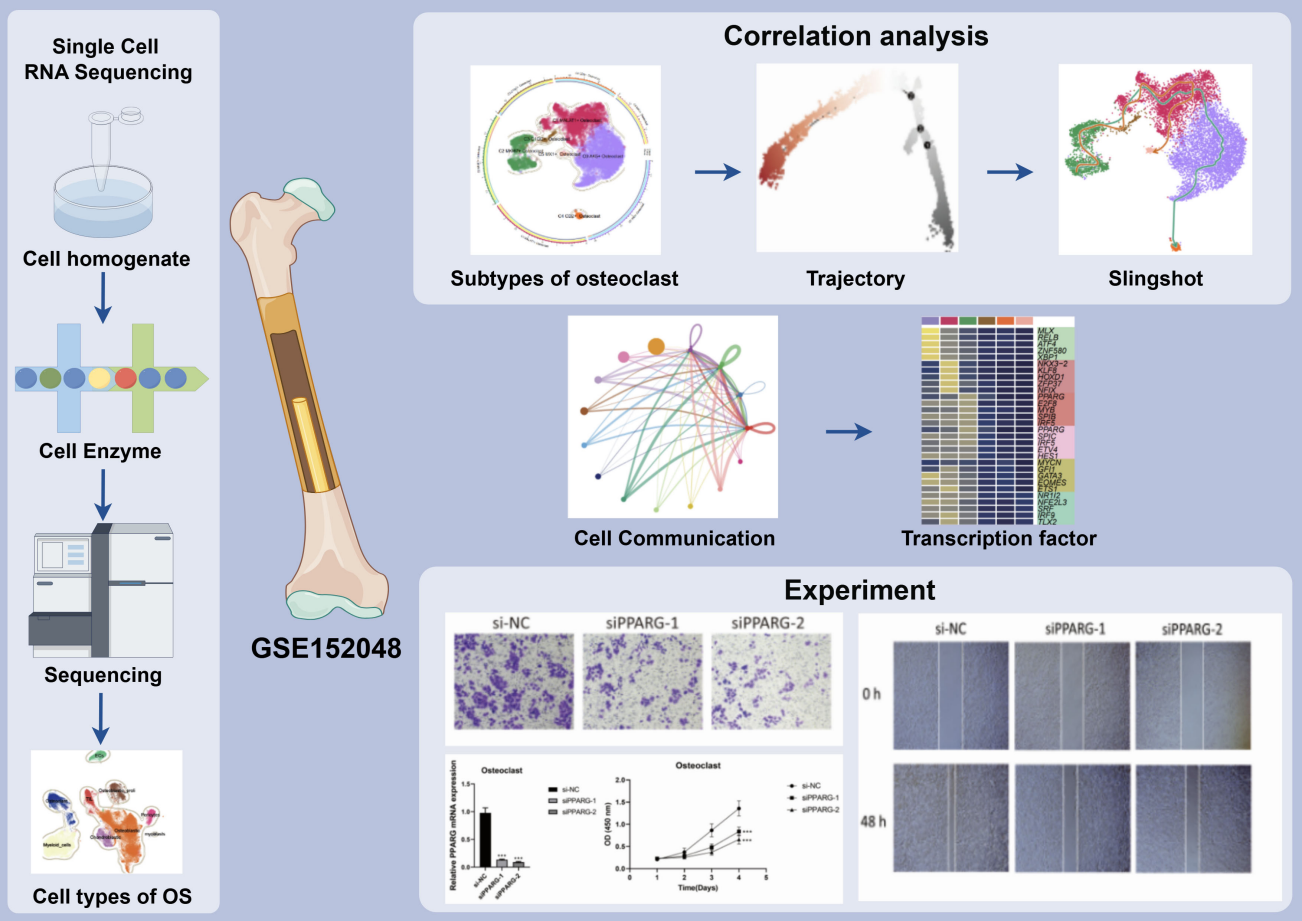
Article flow chart. ScRNA-seq analysis of 10,147 osteoclasts in osteosarcoma tissues showed that C2 MKI67 + Osteoclast was a key subgroup and expressed the characteristics of high proliferation and differentiation, which was verified by cell trajectory. In addition, we also found that the signaling pathway APP and the transcription factor PPARG may provide new ideas for treatment of OS. The results showed that the related targets played an important role in the proliferation, activation, and migration of osteoclasts.

**Figure 2:**
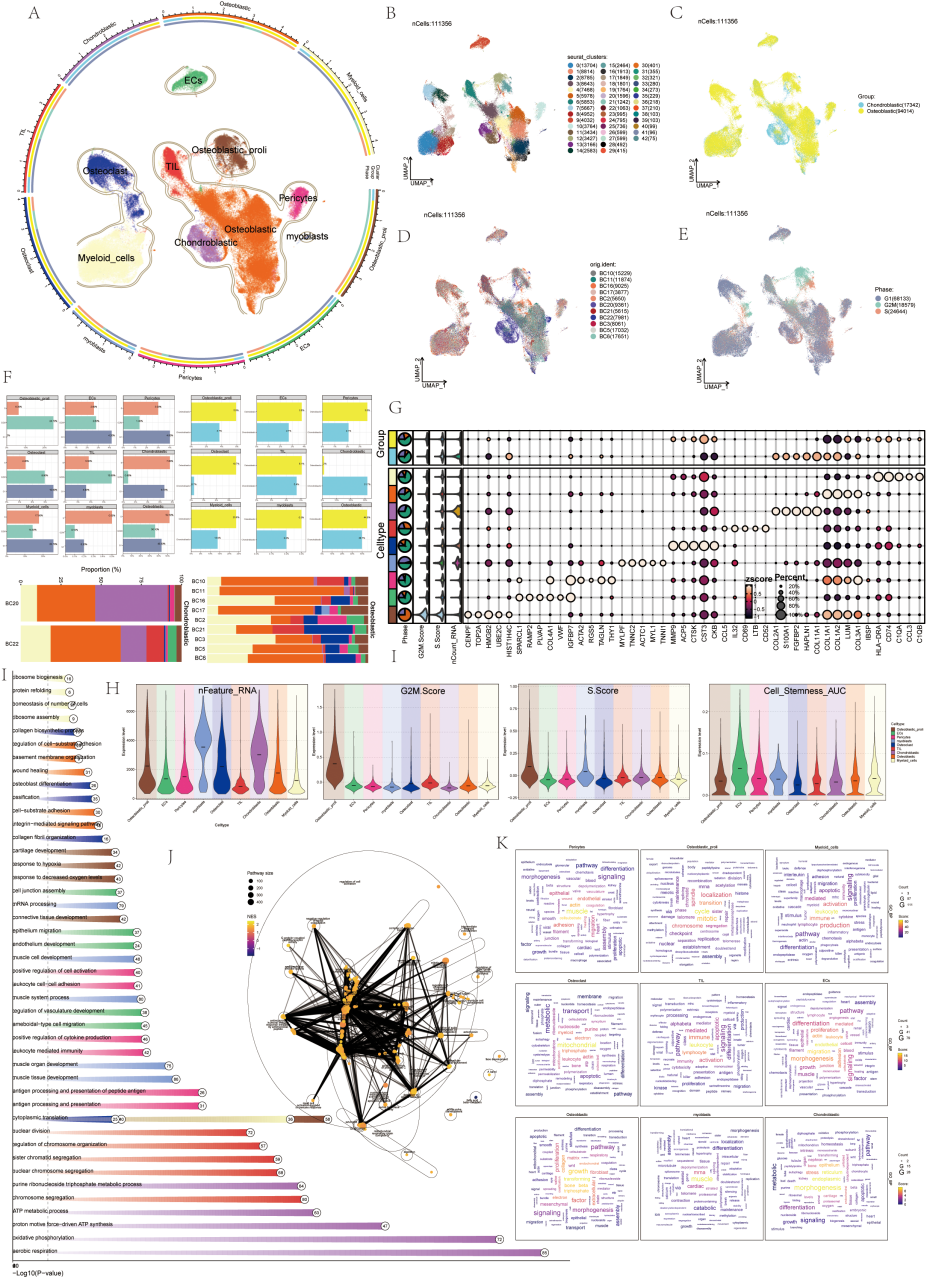
ScRNA-seq revealed the tumor microenvironment of OS. **(A)** UMAP plots depicted the distribution of 9 cell types in OS, with each point representing a cell. Similar cell types clustered together, including: ECs, Osteoblastic proliferating cells, Osteoclasts, Myeloid cells, Chondroblastic, Osteoblastic, Pericytes, myoblasts, TIL. **(B)** UMAP plots showed 43 cell clusters in OS patients and the number of cells in each cluster. **(C)** UMAP plots categorized OS patients into two groups: osteoblastic (yellow) and chondroblastic (blue). **(D)** UMAP plots displayed the sample sources of OS patients, including: BC10, BC11, BC16, BC17, BC2, BC20, BC21, BC22, BC3, BC5, BC6. **(E)** UMAP plots illustrated cell cycles during OS progression, including: G1 (gray), G2M (green), and S (red). **(F)** Histograms depicted the percentage of 9 cell types in the cell cycles, groups, and samples. **(G)** The bubble diagram displayed that the top 10 marker genes in different cell types. **(H)** Violin plots showed S.core, G2M.score, Cell-Stemness-AUC, cell_Stemness_AUC, and nFeature-RNA of 9 cell types. **(I)** The GOBP enrichment analysis bar chart showed biological processes related to 9 cell types. **(J)** The enrichment network diagram displayed the enrichment of all differential genes in various cell subsets in OS. **(K)** Cloud charts displayed the GO-BP enrichment analysis results of various cell subsets in OS.

First of all, we showed the flow chart of this article as shown in **Figure 1**. The data of this study were derived from sample number GSE152048. We analyzed it by scRNA-seq. Visualized using UMAP plots, OS was categorized into nine cell types: ECs, Osteoblastic proliferating cells, Osteoclasts, Myeloid cells, Chondroblastic, Osteoblastic, Pericytes, Myoblasts, and TIL (**Figure 2A**). A total of 43 seurat clusters were identified (**Figure 2B**), and the sample origins included BC10 (15229), BC11 (11874), BC16 (9025), BC17 (3877), BC2 (5650), BC20 (9361), BC21 (5615), BC22 (7981), BC3 (8061), BC5 (17032), and BC6 (17651) (**Figure 2D**). The dataset comprised two distinct groups:osteoblastic OS (94014) and chondroblastic OS (17342), with the latter primarily located in the lower left region (**Figure 2C**). Notably, in the cell cycle distribution, G2M and S phases were concentrated in the upper right and left sides, indicating rapid cell proliferation and a highly differentiated state (**Figure 2E**). In addition, bar diagrams illustrate the cell cycle, group and sample of the nine cell subtypes Osteoblastic was highly expressed in both tissue types, whereas Osteoclast was predominantly found in OS, with a proportion second only to Osteoblastic and Myeloid cells (**Figure 2F**). The bubble diagram showed the top 5 marker genes of each cell type in OS (**Figure 2G**). Violin plots displayed the expression of S.Score, G2M.Score, Cell_Stemness_AUC, and nFeature_RNA among the nine cell types. Results showed higher S.Score and G2M.Score in Osteoblastic proliferating cells, correlating with its proliferative phase. ECs exhibited the highest Cell_Stemness_AUC, while Myeloid cells, Chondroblastic, and Osteoclasts showed strong performances in nFeature_RNA (**Figure 2H**). Finally, GOBP enrichment analysis for the nine cell types highlighted their enriched pathways: chromosome segregation, ameboidal-type cell migration, muscle organ development in myoblasts, cytoplasmic translation, aerobic respiration, leukocyte-mediated immunity, and so on (**Figures 2I-K**).

### Annotation and enrichment analysis of osteoclasts

**Figure 3:**
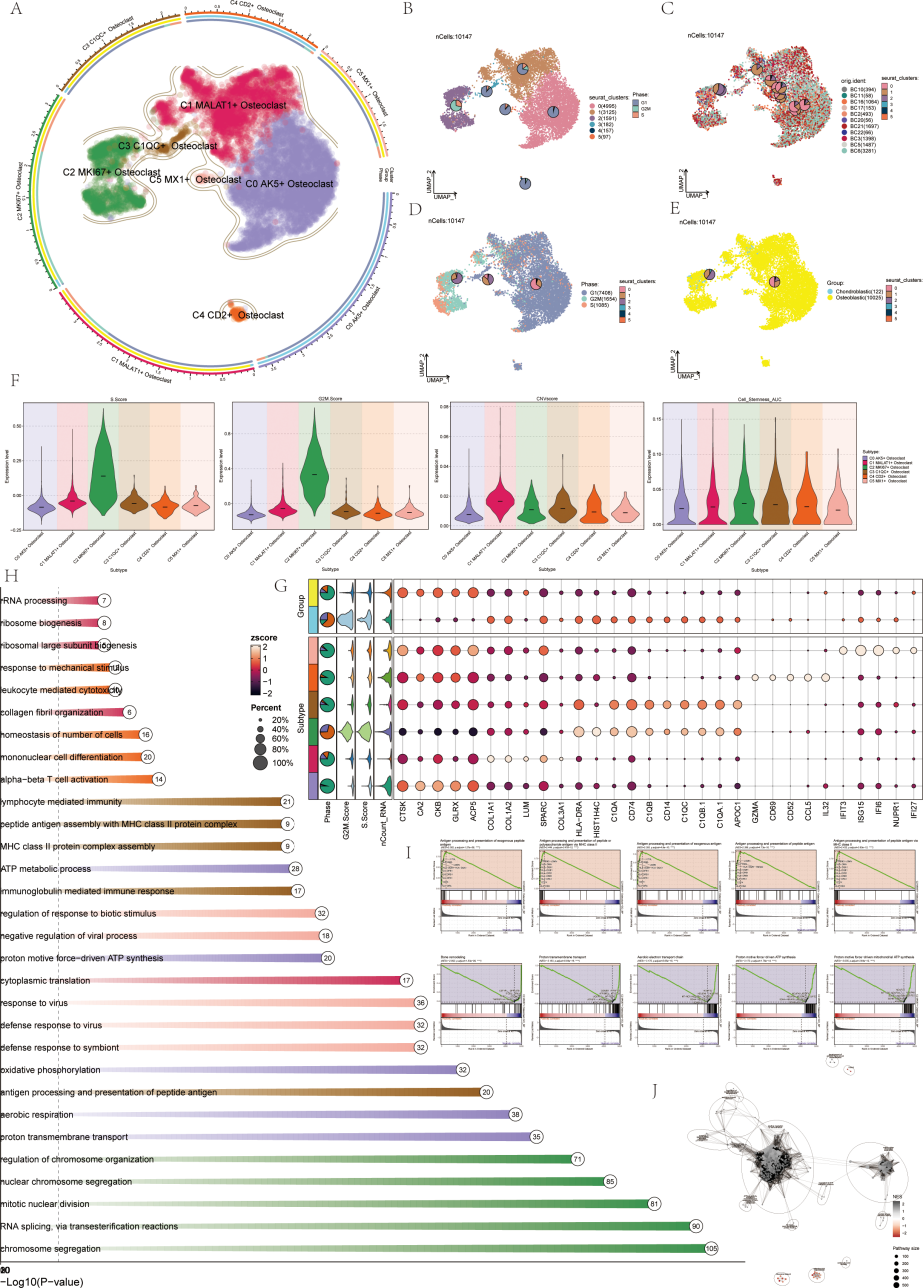
Visualization of osteoclasts. **(A)** UMAP plots showed the distribution of 6 subgroups of osteoclasts, including: C0 AK5+ Osteoclast (purple), C1 MALAT1+ Osteoclast (red), C2 MKI67+ Osteoclast (green), C3 C1QC+ Osteoclast (brown), C4 CD2+ Osteoclast (orange), C5 MX1+ Osteoclast (pink). **(B)** UMAP plots depicted 6 seurat clusters of osteoclasts and their cell numbers. **(C)** UMAP plots displayed the sample sources and cell numbers of osteoclasts in OS patients. **(D)** UMAP plots illustrated the distribution of osteoclasts in cell cycles and their cell numbers. **(E)** UMAP plots showed the distribution and cell numbers of osteoclasts in osteoblastic (yellow) and chondroblastic (blue) groups. **(F)** Violin plots displayed characteristics of 6 osteoclasts subgroups, including S.core, G2M.score, CNVscore, and Cell-Stemness-AUC. **(G)** Bubble plots showed differential expression of top 5 marker genes among 6 osteoclasts subgroups. Bright yellow indicated stronger specificity, with size representing the percentage of gene expression within subgroups. **(H)** Enrichment analysis diagram displayed biological processes related to 6 osteoclasts subgroups in GOBP enrichment analysis. **(I)** GSEA enrichment analysis plots showed 5 upregulated and 5 downregulated enrichment entries associated with the C2 subgroup. **(J)** The enrichment network diagram showed the enrichment of osteoclast subsets.

Previous experience showed that osteoclasts were a key link in the formation and progression of OS, and their performance in OS was also highly specific. Therefore, this study mainly analyzed osteoclasts from a single-cell perspective. Visual analysis of Osteoclasts via UMAP plots identified six seurat clusters (**Figure 3B**) and six cell subtypes: C0 AK5+ Osteoclast, C1 MALAT1+ Osteoclast, C2 MKI67+ Osteoclast, C3 C1QC+ Osteoclast, C4 CD2+ Osteoclast, and C5 MX1+ Osteoclast (**Figure 3A**). The sample origins included BC10 (394), BC11 (58), BC16 (1064), BC17 (153), BC2 (493), BC20 (56), BC21 (1697), BC22 (66), BC3 (1398), BC5 (1487), and BC6 (3281) (**Figure 3C**). G1 phase was primarily on the right, while G2M and S phases were on the left, notably in the C2 MKI67+Osteoclast cluster, indicating high proliferation and differentiation (**Figure 3D**). The samples were divided into Chondroblastic OS (122) and Osteoblastic OS (10025) (**Figure 3E**). Violin plots showed C2 MKI67+ Osteoclast exhibited high S Score, G2M Score, CNVscore, and Cell_Stemness_AUC (**Figure 3F**), suggesting its crucial role in osteoclast generation and differentiation. Bubble plots highlighted the marker genes of the six osteoclast cell subtypes: C0 AK5+ Osteoclast (CTSK, CA2, CKB, GLRX, ACP5), C1 MALAT1+ Osteoclast (COL1A1, COL1A2, LUM, SPARC, COL3A1), C2 MKI67+ Osteoclast (HLA-DRA, HIST1H4C, C1QA, CD74, C1QB), C3 C1QC+ Osteoclast (CD14, C1QC, C1QB.1, C1QA.1, APOC1), C4 CD2+ Osteoclast (GZMA, CD69, CD52, CCL5, IL32), and C5 MX1+ Osteoclast (IFIT3, ISG15, IFI6, NUPR1, IFI27) (**Figure 3G**). Enrichment analysis displayed pathways for each osteoclast subtype (**Figure 3H**). GSEA enrichment analysis for C2 MKI67+ Osteoclast showed upregulation in pathways like antigen processing and presentation of exogenous peptide antigen and so on (**Figure 3I**). The enrichment network diagram further showed the enrichment of osteoclast subsets (**Figure 3J**).

### Visualization of pseudo-sequential analysis of osteoclasts in OS

**Figure 4:**
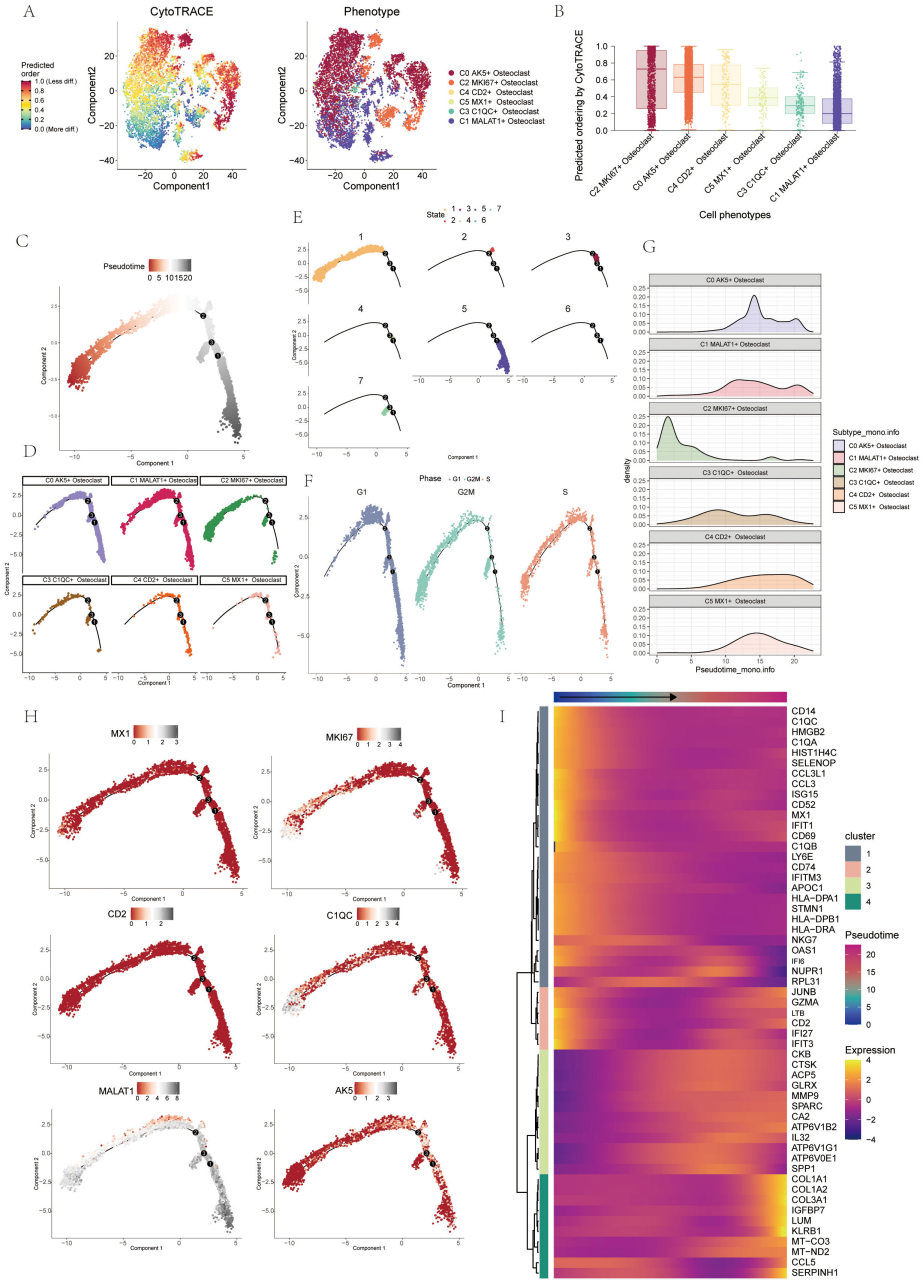
Pseudo-sequential analysis of osteoclasts was demonstrated through CytoTRACE and monocle. **(A, B)** Visual representation of CytoTRACE results for osteoclasts predicted their differentiation sequence ranked from high to low: C2-C0-C4-C5-C3-C1. **(C)** Pseudo-sequential analysis of osteoclasts. Colors transitioned from red to gray, indicating increasing differentiation. **(D)** Pseudotemporal differentiation trajectories of 6 osteoclasts subgroups. **(E)** Pseudotemporal differentiation trajectories of osteoclasts across states. **(F)** Pseudotemporal differentiation trajectories of osteoclasts across cell cycles. **(G)** Ridge plot displayed the differentiation sequence of 6 osteoclasts subgroups. **(H)** Pseudotemporal differentiation trajectories of genes named for 6 osteoclasts subgroups (MX1, MKI67, CD2, C1QC, MALAT1, AK5). **(I)** Heatmap displayed dynamic changes in osteoclasts-related genes throughout the differentiation process.

CytoTRACE software was used to analyze and visualize the differentiation of osteoclasts, and the differentiation potential of osteoclasts was predicted to be C2-C0-C4-C5-C3-C1 from high to low. Among these, C2 MKI67+ Osteoclasts demonstrated the highest stemness and differentiation potential (**Figures 4A, B**). Monocle2 computed the overall differentiation sequence, moving from the lower left to the upper right to differentiation point 2, then branching into two paths from differentiation point 3 (**Figure 4C**). The differentiation sequence of osteoclasts across different states, cell subtypes, and cell cycles was confirmed. The analysis revealed that C2 and C3 subtypes were positioned at the starting phase, C1 subtypes were evenly distributed throughout, while C0, C4, and C5 subtypes were concentrated near the terminal phase (**Figure 4D**). State differentiation aligned with the overall sequence, with C2 primarily in state 1, indicating the beginning of the time trajectory (**Figure 4E**). Cell cycle differentiation showed G2M and S phases at the start, mainly in the C2 subtype, indicating high cell division and proliferation (**Figure 4F**). Ridge plots displayed the differentiation sequence, with C2 MKI67+ Osteoclast at the initial position (**Figure 4G**). Named gene trajectory analysis also showed MKI67 at the beginning of the pseudotime trajectory (**Figure 4H**). In addition, the dynamic changes of osteoclast-related genes during the whole differentiation process were shown by a heat map (**Figure 4I**).

**Figure 5:**
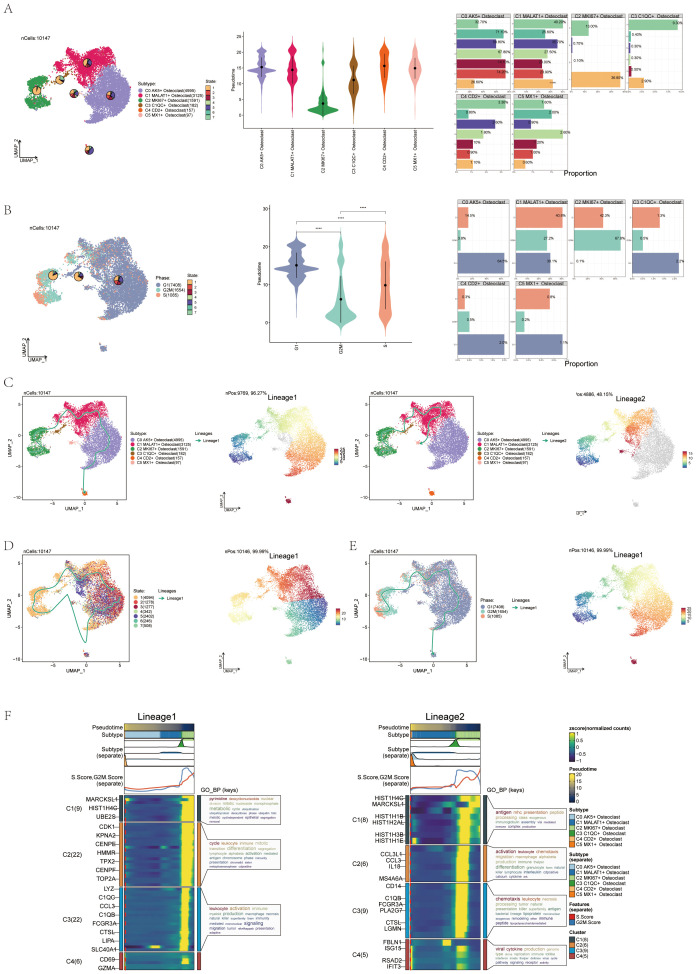
Pseudotemporal differentiation process of osteoclasts was demonstrated through slingshot. **(A)** UMAP, violin plot and bar graphs illustrated the differentiation sequence and state distribution among osteoclast subgroups, identifying the sequence as C2-C3-C5-C1-C0-C4. **(B)** UMAP, violin plot and bar graphs depicted the cell cycles in osteoclast subgroups. ****p < 0.0001. **(C)** UMAP plots revealed two differentiation trajectories (lineage 1 and lineage 2) of osteoclasts along with their respective differentiation sequences. **(D)** UMAP plots depicted the differentiation sequences of lineage1 osteoclast trajectorie across different states. **(E)** UMAP plots illustrated the differentiation sequences of lineage1 osteoclast trajectorie across various cell cycles. **(F)** The heatmaps presented the GO-BP enrichment analysis, showcasing the biological processes associated with the two osteoclast differentiation trajectories.

UMAP, violin, and bar plot validated this order: C2-C3-C5-C1-C0-C4, state 1 accounting for 36.8% and state 7 for 13% in C2 (**Figure 5A**). UMAP, violin, and bar plot confirmed G2M phase at 67.8% and S phase at 42.3% in the C2 subtype (**Figure 5B**). Slingshot pseudotime analysis was performed on 6 subgroups, showing two lineages: lineage1 (C2-C3-C1-C0-C4) and lineage2 (C2-C3-C1-C5), with C2 MKI67+ Osteoclast at the start of both (**Figure 5C**). State differentiation showed that the C0 subtype mainly corresponded to state 2, while the C2 subgroup mainly corresponded to state 1 (**Figure 5D**). UMAP plots illustrated the differentiation sequences of lineage 1 osteoclast trajectorie across various cell cycles (**Figure 5E**). These analyses identified C2 MKI67+ Osteoclast as the key subtype. Enrichment analysis for cell subtypes showed pyrimidine, deoxyribonucleotide, cycle, leukocyte activation, antigen chemotaxis, and other entries enriched mainly in the C2 subtype in both differentiation trajectories (**Figure 5F**).

### Cell-cell interaction in OS

**Figure 6:**
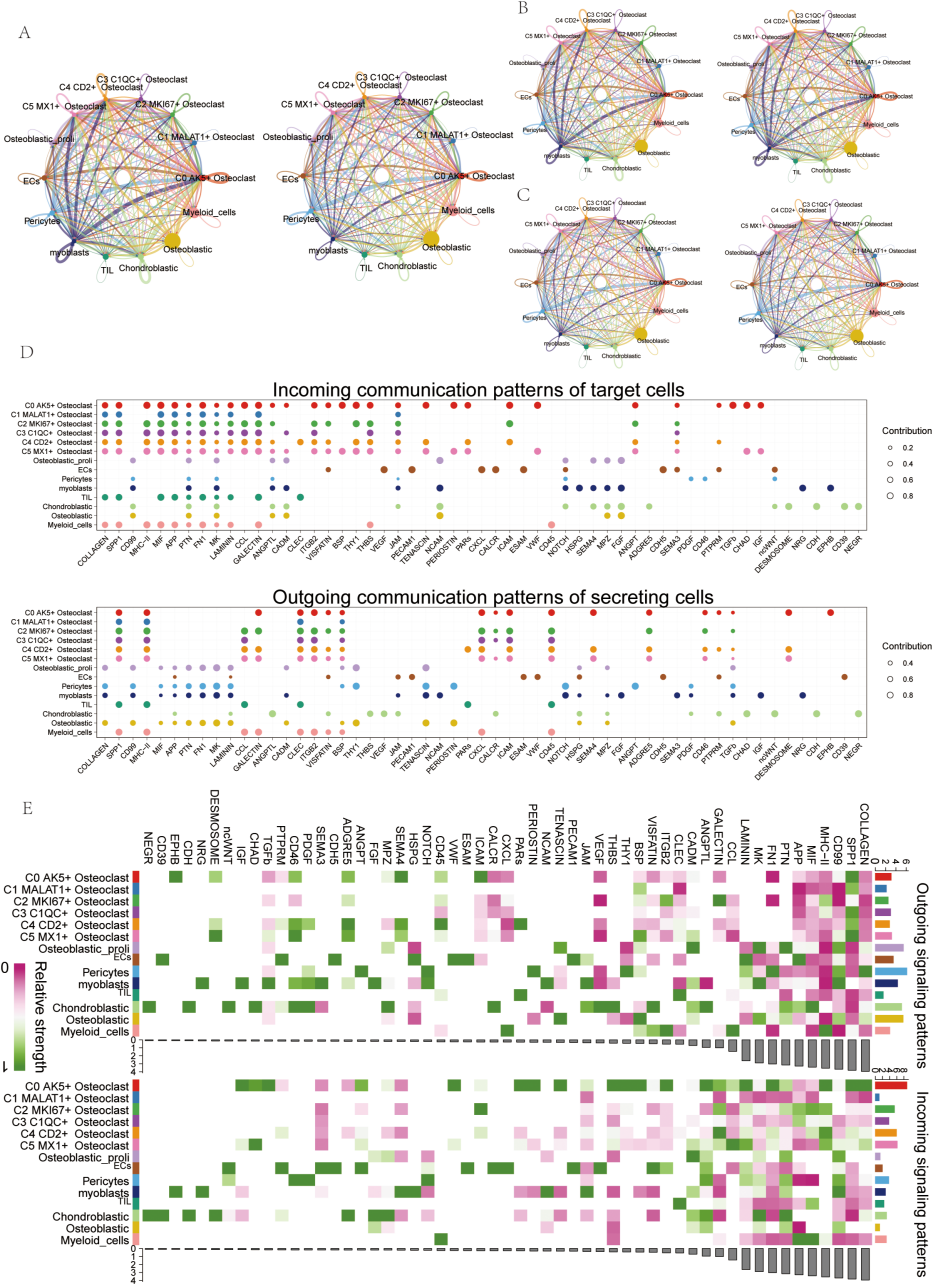
Cell communication visualization in OS. **(A)** Circle plots depicted the number and weight of interactions among 9 cell types in OS. **(B, C)** Circle plots depicted the number (top) and weight (down) of interactions of osteoclasts with other cell types. **(D)** Bubble plots displayed incoming and outgoing communication patterns of target and secreting cells, further illustrating cell interactions between osteoclasts and other cell types. **(E)** Heatmap showed input and output signal strengths of interactions among all cell types.

Using interaction analysis, we established a cellular communication network among various cell types in OS, including ECs, Osteoblastic proliferating cells, Myeloid cells, Chondroblastic, Osteoblastic, Pericytes, Myoblasts, TIL, and six osteoclast subtypes (**Figure 6A**). The interaction number (**Figure 6B**) and strength (**Figure 6C**) were depicted in circle plots, and the thickness of the connecting lines was correlated with the expression of quantity and strength to identify key input and output signals related to the six osteoclast subtypes, we illustrated the incoming and outgoing communication patterns through bubble plots, predicting them. Cell subsets could act as secretory cells (signal senders) releasing cytokines or ligands or as target cells (signal receivers) when their receptors were targeted by ligands released by other cells. We found that the six osteoclast subtypes, when acting as secretory cells, released cytokines or ligands such as APP, GALECTIN, CLEC, ITGB2, etc. As target cells, their receptors included COLLAGEN, APP, and MIF (**Figure 6D**). In addition, according to the heat map, in incoming signaling patterns, the APP signaling pathway was strongly expressed in some osteoclast subsets, while in outgoing signaling patterns, the APP signaling pathway was mainly related to ECs and chondroblastic (**Figure 6E**).

### APP signaling pathway in osteoclasts and APP-CD74 ligand-receptor pair

**Figure 7:**
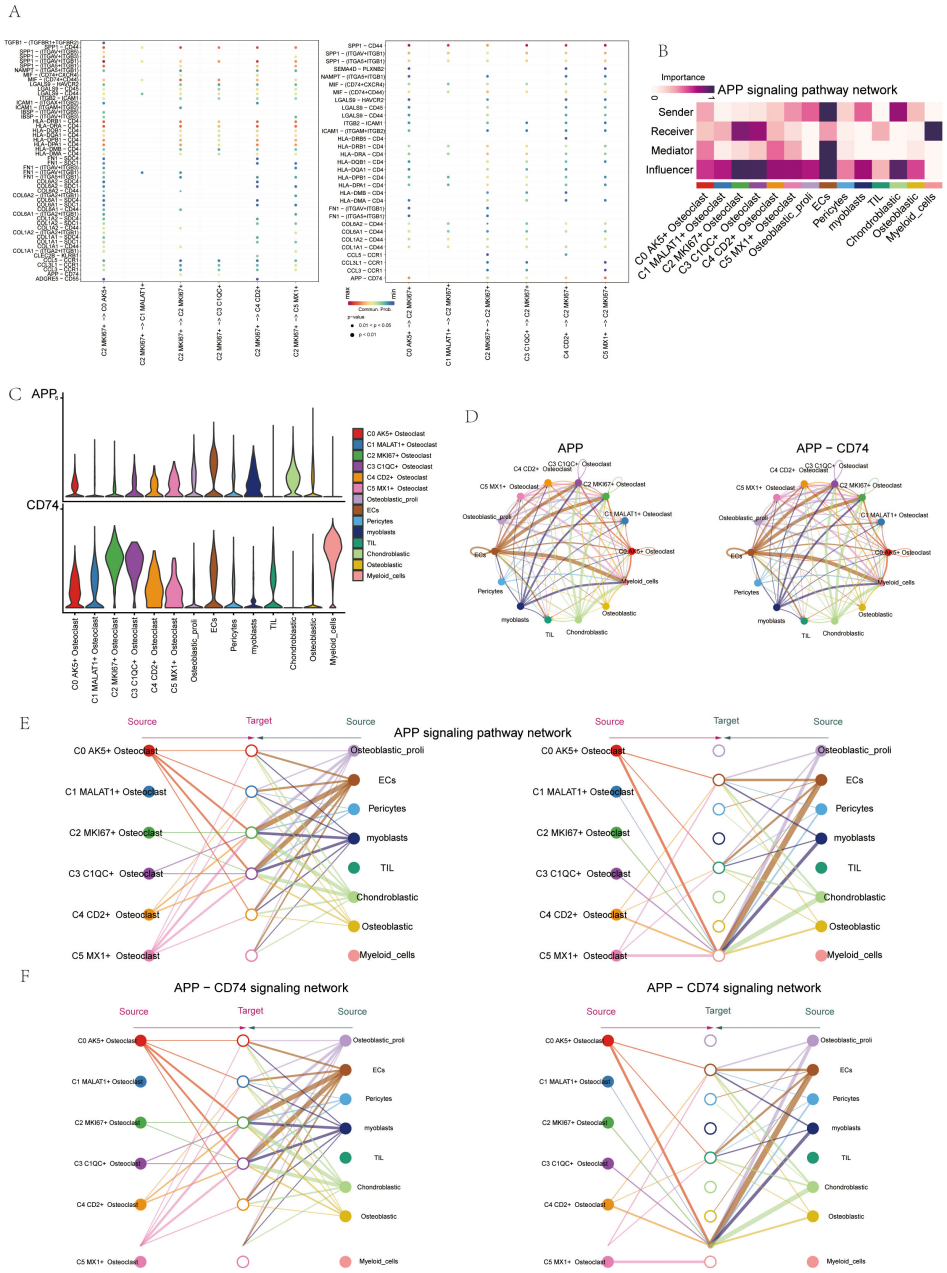
APP signaling pathway and APP-CD74 Ligand-Receptor Pair. **(A)** Bubble plots displayed the expression of receptor pairs when the C2 subgroup served as either source (left) or target (right), and when other subgroups served as targets (left) or sources (right). **(B)** Heatmap showed centrality scores of cell types in the APP signaling pathway network. **(C)** Violin plots showed cell interactions in the APP signaling pathway network. **(D)** Circle plots displayed cell interactions among the APP signaling pathway and the APP-CD74 ligand-receptor pair among osteoclast subgroups. **(E, F)** The hierarchical diagram showed interactions among cells in the APP signaling pathway and the APP-CD74 ligand-receptor pair, with thicker lines indicating stronger interactions.

In order to further study the interaction between osteoclasts and tumor cells, we screened out the pathways that interact with tumor cells. We found that the specific signaling pathway in cell interaction was the APP signaling pathway. To explore the mode of action of the APP signaling pathway, bubble plots showed high expression of SPP1-CD44, APP-CD74, and HLA-DRA-CD4 ligand-receptor pairs when osteoclasts acted as secretory or target cells (**Figure 7A**). Then we performed a “centrality measurement”, which showed the relative importance of each cell type as a sender, receiver, mediator, and influencer in the APP signaling pathway network through heatmap. The results showed that in the APP signaling pathway, the C2 subgroup had the higher expression as a receiver and influencer (**Figure 7B**). The violin plot showed cell-cell interactions and found that the C2 subgroup was highly expressed on CD74 and the Chondroblastic was highly expressed on APP (**Figure 7C**). Then, the interaction between the cells in the APP signaling pathway and the interaction between the cell subsets in the APP-CD74 ligand-receptor pair with specificity were shown by the circle plots (**Figure 7D**). We knew that all cell types were the source cells of the APP signaling pathway. We needed to select specific cell types as potential target cells and visualize the potential targets of APP released by different cell types through hierarchical maps (**Figure 7E**). The APP-CD74 ligand-receptor pair interpretation of the layer diagram was similar to **Figure 7F**.

### Oxidative phosphorylation metabolic pathway in osteoclasts

**Figure 8:**
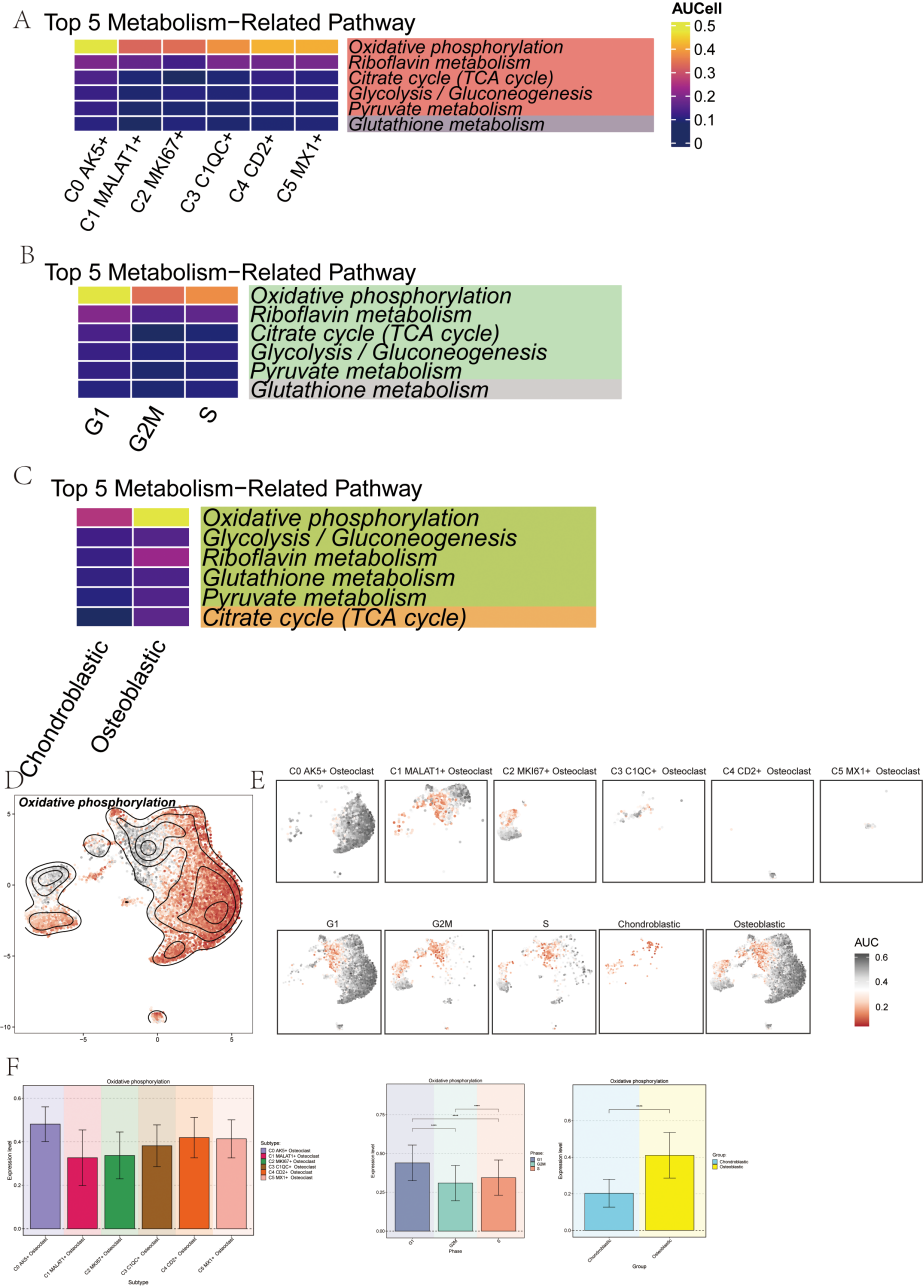
Metabolic pathways related to osteoclast subsets. **(A)** The heatmap showed the AUCell score of the top 5 metabolic pathway of osteoclast subsets. **(B)** The heatmap showed the AUCell scores of top 5 metabolism-related pathways in different cell cycles (G1, G2M, and S phases). **(C)** The heat map showed the AUCell scores of the top 5 metabolic-related pathways in different groups (chondroblastic, osteoblastic). **(D, E)** The UMAP plot and the faceted graphs showed the distribution of AUCell values of oxidative phosphorylation in 6 osteoclast subsets, 2 groups, and 3 cell cycles. **(F)** The bar plots showed differences in the expression levels of oxidative phosphorylation in 6 osteoclast subsets, 2 groups, and 3 cell cycles. ****p < 0.0001.

We further explored cell metabolism to identify metabolic pathways closely associated with osteoclasts. First, we calculated and visualized the AUCell scores of the top 5 metabolic pathways across six osteoclast subsets. These pathways included oxidative phosphorylation, riboflavin metabolism, the citrate cycle (TCA cycle), glycolysis/gluconeogenesis, and pyruvate metabolism (**Figure 8A**). Next, we presented the AUCell scores of the top 5 metabolism-related pathways across different cell cycles (**Figure 8B**) and groups (**Figure 8C**). Our analysis revealed that oxidative phosphorylation was the metabolic pathway most strongly associated with osteoclasts. Using UMAP and facet diagrams, we displayed the distribution of AUCell values (**Figures 8D, E**) for oxidative phosphorylation across different osteoclast subsets, groups, and cell cycles.

Finally, bar plots indicated that oxidative phosphorylation was highly expressed in several osteoclast subsets and exhibited higher activity during the osteoblastic and G1 phases (**Figure 8F**).

### Gene regulatory network of osteoclast subgroups

**Figure 9:**
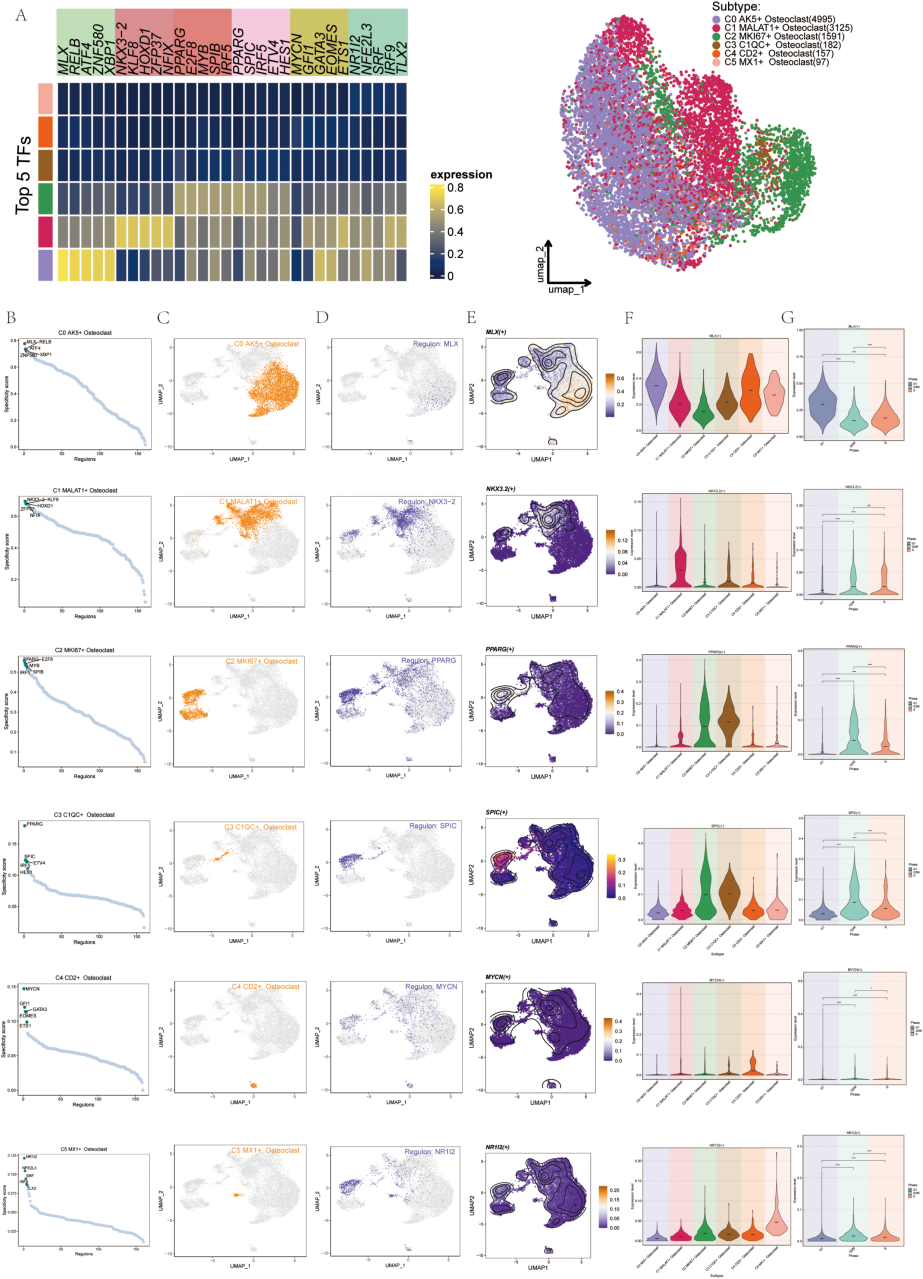
Gene regulatory network (GRN) of Osteoclast subgroups. **(A)** The heatmap displayed the expression levels of the top 5 transcription factors (TFs) across each osteoclast subgroup. The UMAP diagram illustrated the distribution patterns of these osteoclast subsets. **(B)** Scatter diagrams showed the distribution of the top 5 regulons (green dots) among the 6 osteoclast subgroups. **(C)** UMAP plots displayed the distribution of these 6 osteoclast subgroups (orange dots). **(D)** UMAP plots displayed the distribution of the most active regulons in each osteoclast subgroup. **(E)** UMAP plots displayed the distribution of the most active TFs in each osteoclast subgroup. **(F)** Violin plots displayed expression of the most active TFs in the 6 osteoclast subgroups. **(G)** Violin plots displayed expression of the most active TFs across cell cycles in the 6 osteoclast subgroups. *p < 0.05, ****p < 0.0001, ns indicated no statistical difference.

To identify core transcription factors (TFs) within the six osteoclast subgroups, we conducted an analysis using PySCENIC. This approach also allowed us to infer the gene regulatory networks specific to each osteoclast subset. Initially, through heatmap analysis, we identified the top 5 TFs within the six osteoclast subgroups, with PPARG, E2F8, MYB, SPIB, and IRF5 prominent in the C2 subgroup (**Figure 9A**). Using pySCENIC software, we ranked regulons based on their regulatory specificity scores (RSS), where a higher RSS indicated the regulon had a closer relationship to the cell type. The scatter diagrams showed the binarized regulon activity scores (RAS) of the major regulators (green dots) in osteoclast subgroups. The top 5 TFs in each subgroup were MLX, RELB, ATF4, XBP1, and ZNF580 in C0; NKX3-2, KLF8, HOXD1, ZFP37, and NFIX in C1; PPARG, E2F8, MYB, SPIB, and IRF5 in C2; PPARG, SPIC, ETV4, IRF5, and HES1 in C3; MYCN, GFI1, GATA3, EOMES, and ETS1 in C4; NR1L2, NFE2L3, SRF, IRF9, and TLX2 in C5 (**Figure 9B**). The most active TFs in each subgroup were MLX, NKX3-2, PPARG, MYCN, and NR1I2. UMAP plots also illustrated the distribution of these six osteoclast subgroups (orange dots) (**Figure 9C**). Additionally, we highlighted the most specific regulons: MLX in the C0 subgroup, NKX3-2 in C1, PPARG in C2, PPARG in C3, MYCN in C4, and NR1L2 in C5. UMAP plots demonstrated the distribution and expression patterns of the most active TFs (**Figures 9D, E**). To further understand their expression variations across subgroups (**Figure 9F**) and cell cycles (**Figure 9G**), violin plots depicted the distribution of MLX primarily in C1 and C4 during the G1 phase, NKX3-2 mainly in C1 during the G2M and S phases, PPARG predominantly in C2 and C3 during the G2M and S phases, MYCN in C4 with less pronounced phase-specific expression, and NR1L2 in C5. In this study, we identified the C2 subgroup as pivotal, with PPARG identified as its most active TF. Numerous studies have associated PPARG with osteoclast differentiation and proliferation.

### TF regulatory modules of osteoclasts

**Figure 10:**
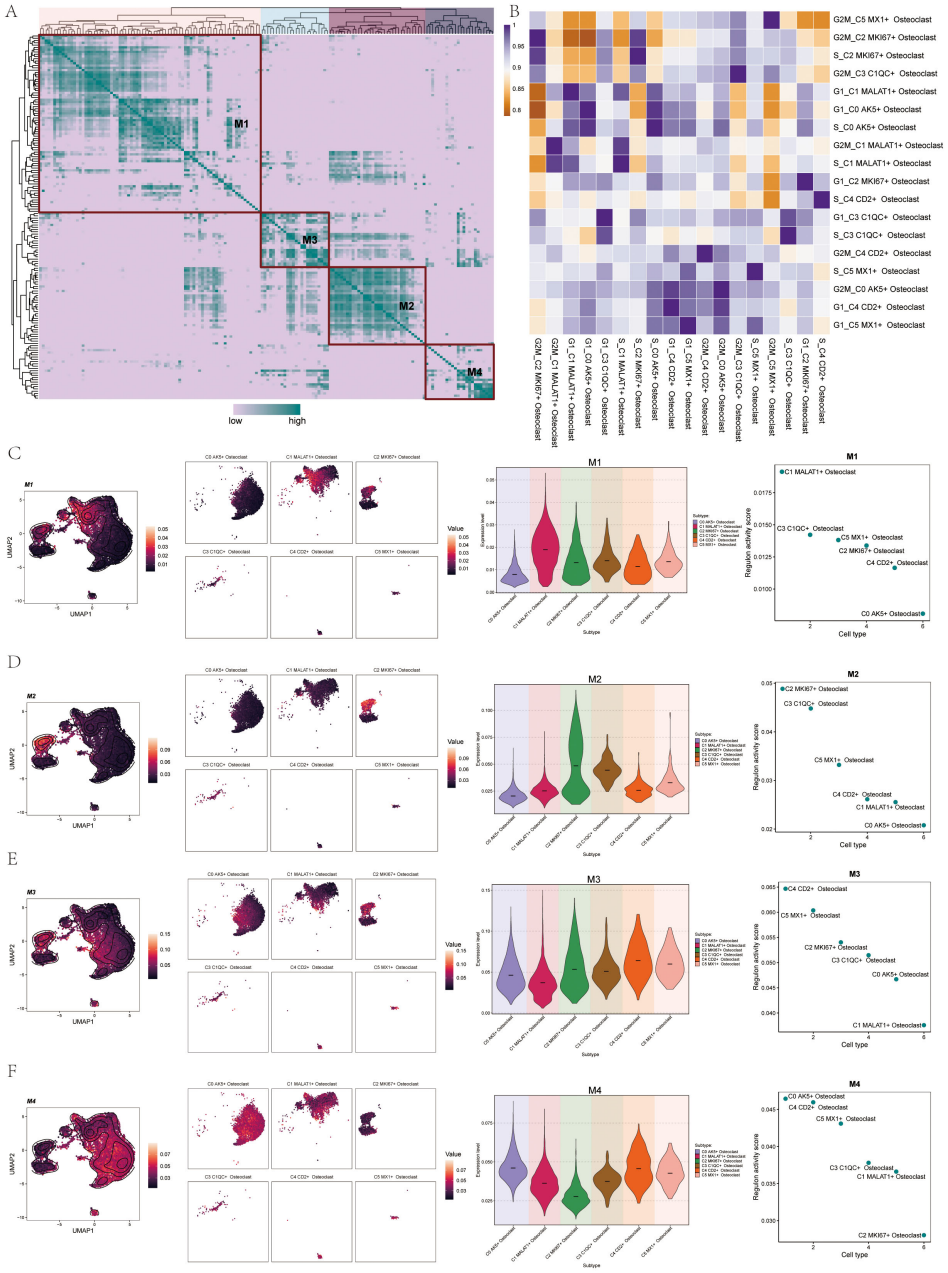
TF regulatory modules of Osteoclasts. **(A)** The heatmap showed the TF regulon modules (M1, M2, M3, and M4) of osteoclast subsets. **(B)** The heatmap displayed the transcriptional regulation activity between osteoclast subsets in different cell cycles. **(C)** UMAP plots and facet graphs showed expression associated with M1, with specific distribution details among osteoclast subgroups. Violin plot detailed the distribution of M1 among osteoclast subgroups by regulon activity score, and scatter plots depicted subgroup sequences. **(D)** UMAP plots and facet graphs showed expression associated with M2, with specific distribution details among osteoclast subgroups. Violin plot detailed the distribution of M2 among osteoclast subgroups by regulon activity score, and scatter plots depicted subgroup sequences. **(E)** UMAP plots and facet graphs showed expression associated with M3, with specific distribution details among osteoclast subgroups. Violin plot detailed the distribution of M3 among osteoclast subgroups by regulon activity score, and scatter plots depicted subgroup sequences. **(F)** UMAP plots and facet graphs showed expression associated with M4, with specific distribution details among osteoclast subgroups. Violin plot detailed the distribution of M4 among osteoclast subgroups by regulon activity score, and scatter plots depicted subgroup sequences.

We found the regulatory module of osteoclast subtypes by the connection specific index (CSI) matrix. We categorized TFs into four main modules (M1, M2, M3, and M4) and mapped these modules onto UMAP plots, initially highlighting M1-related expression (**Figure 10A**). In order to understand the correlation of transcriptional regulation activity between osteoclast subsets in OS, we analyzed osteoclast subsets in different cell cycles. The results were shown in the figure (**Figure 10B**). Using facet graphs, we detailed the M1 distribution across various osteoclast subgroups, noting the highest expression in C1 and the lowest in C0. Validation through violin plot confirmed these findings, with regulatory activity scores sorting cell subgroups as follows: C1, C3, C5, C2, C4, and C0 (**Figure 10C**). We further illustrated the distribution of M2 (**Figure 10D**), M3 (**Figure 10E**), and M4 (**Figure 10F**) modules on UMAP plots and facet graphs, providing additional confirmation through violin plots and scatter plots. Based on the regulatory activity score, the expression levels of each subgroup in M2 were C2, C3, C5, C4, C1 and C0, respectively. The expression levels of each subgroup in M3 were C4, C5, C2, C3, C0 and C1. In M4, followed by C0, C4, C5, C3, C1, C2.

**Figure 11:**
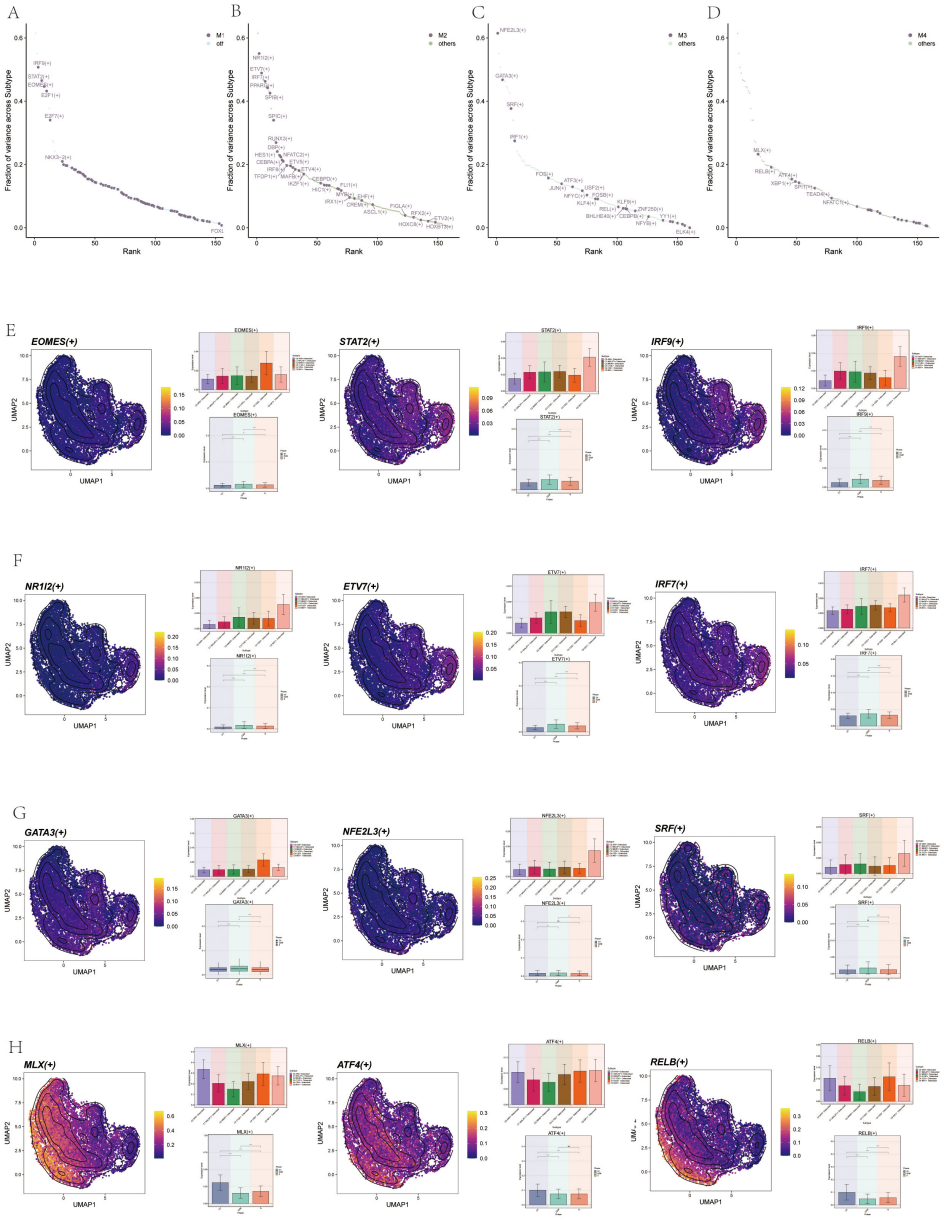
Visualization of TFs in Osteoclasts. **(A)** Scatter plot ranked TFs in M1 by variance fraction. **(B)** Scatter plot ranked TFs in M2 by variance fraction. **(C)** Scatter plot ranked TFs in M3 by variance fraction. **(D)** Scatter plot ranked TFs in M4 by variance fraction. **(E)** UMAP plots displayed expression of the top 3 TFs (EOMES, STAT2, IRF9) in M1, with histograms showing their expression across osteoclast subgroups and cell cycles. **(F)** UMAP plots displayed the expression of the top 3 TFs (NR1I2, ETV7, and IRF7) in M2, with histograms showing their expression.across osteoclast subgroups and cell cycles. **(G)** UMAP plots displayed expression of the top 3 TFs (NFE2L3, GATA3, and SRF) in M3, with histograms showing their expression across osteoclast subgroups and cell cycles. **(H)** UMAP plots displayed expression of top 3 TFs (MLX, RELB, and ATF4) in M4, with histograms showing their expression across osteoclast subgroups and cell cycles.

We sorted the TFs, with the top 5 TFs in M1 being IRF9, STAT2, EOMES, E2F1, and E2F7 (**Figure 11A**). In M2, the top 3 TFs were NR1I2, ETV7, IRF7, PPARG, and SPIB (**Figure 11B**). In M3, they were NFE2L3, GATA3, SRF, IRF1, and FOS (**Figure 11C**). In M4, they were MLX, RELB, ATF4, XBP1, and SPI1 (**Figure 11D**). We then analyzed the top 3 TFs in each module, showing the distribution of them in each subgroup on UMAP plots, and their expression in different cycles was shown in the violin diagram. For instance, the distribution and expression of EOMES, STAT2, and IRF9 in osteoclast subsets in M1 were demonstrated (**Figure 11E**). Similarly, the distribution and expression of NR1I2, ETV7, and IRF7 in each subgroup and cell cycle in M2 (**Figure 11F**). The distribution and expression of NFE2L3, GATA3, and SRF in M3 (**Figure 11G**). Finally, the distribution and expression of MLX, RELB, and ATF4 in M4 (**Figure 11H**).

### Silencing PPARG inhibited osteoclast proliferation and migration

**Figure 12:**
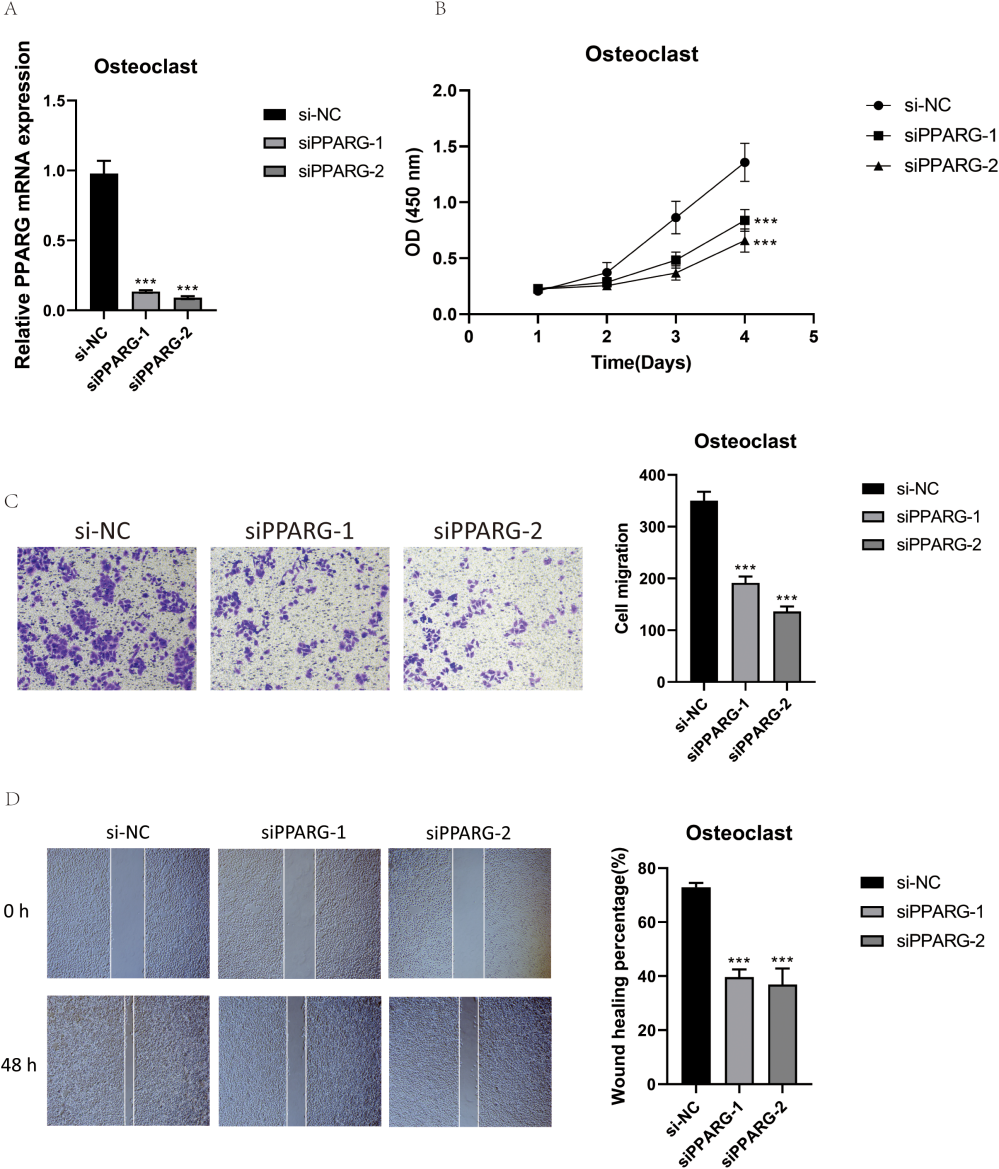
Validation of PPARG *In Vitro* Experiments. **(A)** RT-qPCR was used to detect the expression level of PPARG in osteoclasts. **(B)** The CCK-8 assay showed that PPARG knockout inhibited osteoclast proliferation, with a significant decrease in cell viability. **(C)** The Transwell assay demonstrated that PPARG knockdown significantly reduced the migration of osteoclasts. **(D)** The scratch assay indicated that PPARG knockdown significantly slowed down osteoclast migration. ***p < 0.001.

To further understand the function of PPARG, we conducted *in vitro* functional assays. First, RT-qPCR was used to detect the expression level of PPARG after transfection to determine the effectiveness of siRNA-mediated PPARG knockdown in osteoclast cell lines. The results showed that knocking down PPARG significantly inhibited its expression in osteoclasts (**Figure 12A**). Next, the CCK-8 assay demonstrated that the proliferation capacity of the two osteoclast groups with PPARG knockdown was significantly reduced, and cell viability markedly decreased compared to the control group (**Figure 12B**). The results of the Transwell experiment showed that the staining area of the PPARG knockdown cell line was significantly smaller than that of the control group, indicating that silencing the PPARG gene slowed down the migration of osteoclasts (**Figure 12C**). In the wound healing assay, the migration rate of the two osteoclast groups with PPARG knockdown was slower, with a statistically significant result (**Figure 12D**).

## Discussion

OS (1) is the most common primary malignant bone tumor, characterized by its high aggressiveness and poor prognosis. This disease primarily affects children and adolescents, making it one of the most prevalent malignancies in this population (4). Despite advancements in treatment, the survival rate for osteosarcoma patients remains concerning: the survival rate for primary osteosarcoma is below 70%, while for metastatic osteosarcoma, it further declines to less than 30% (10). Currently, osteosarcoma treatment primarily relies on multidisciplinary approaches, including surgery, chemotherapy, and emerging targeted therapies. In recent years, significant progress has been made in research on targeted therapies for osteosarcoma. However, due to the high drug resistance of osteosarcoma, improving patient survival remains a pressing challenge.

Osteoclasts, as the only cells with bone-resorbing capabilities, primarily mediate the destruction of inorganic bone components and the degradation of collagen through the secretion of acidic substances and proteolytic enzymes (71). The bone-resorbing activity of osteoclasts plays a critical role in both primary and metastatic osteosarcoma. Additionally, the interactions between osteoclasts, osteoblasts, and malignant osteosarcoma cells form a vicious cycle that not only disrupts bone homeostasis but also promotes the progression of osteosarcoma. Osteoclasts will be a key target for the treatment of OS (33, 72). Therefore, this study aims to utilize scRNA-seq technology to uncover the characteristics and functions of highly heterogeneous osteoclast subpopulations in osteosarcoma, track their dynamic changes during tumor progression, and precisely identify key signaling pathways and transcription factors in osteosarcoma. This provides novel insights into the feasibility of targeting osteoclasts for osteosarcoma treatment and offers a scientific basis for developing personalized treatment plans for patients. Firstly, in order to reveal the heterogeneity of osteoclasts in OS and further understand the molecular characteristics of osteoclasts, we subdivided them into 6 subgroups. Each osteoclast subpopulation was named after its highest-expressing gene: C0 AK5+ Osteoclast, C1 MALAT1+ Osteoclast, C2 MKI67+ Osteoclast, C3 C1QC+ Osteoclast, C4 CD2+ Osteoclast, and C5 MX1+ Osteoclast. The analysis of these subgroups revealed that C2 MKI67+ Osteoclasts exhibited the highest expression levels during the S phase and G2/M phase, along with elevated cell stemness AUC scores. Notably, integrating Monocle, CytoTRACE, and Slingshot analyses highlighted that this subgroup had a higher CytoTRACE score and was positioned at the early stage of the cell differentiation trajectory. Additionally, we observed that the C2 MKI67+ Osteoclasts subgroup demonstrated significant activity and was in a phase of high proliferation and differentiation, closely associated with the generation and differentiation of a large number of Osteoclasts. The naming gene for this subpopulation was MKI67. Studies have shown that MKI67 is an antigen identified by the monoclonal antibody Ki-67, a cell proliferation-associated protein encoded by the MKI67 gene. It is predominantly expressed in proliferating cells and widely used in clinical practice. Research further indicates that Ki-67 (MKI67), as a vital prognostic marker, has been extensively applied in the identification of various cancers, including breast cancer, gastric cancer, cervical cancer, and lung cancer (5, 73–81).

Furthermore, studies had shown that Ki-67 levels were closely related to the proliferative activity and malignancy of OS. The remarkable proliferation and differentiation capacity of C2 MKI67+ osteoclasts is closely associated with this gene. Given the high biological activity of this subgroup, we speculate that it possesses strong bone resorption capabilities, potentially leading to bone tissue destruction and influencing the onset and progression of osteosarcoma.

Enrichment analysis of the C2 MKI67+ Osteoclast subgroup revealed its close association with processes such as chromosome segregation, mitotic nuclear division, sister chromatid segregation, mitotic sister chromatid segregation, and nuclear chromosome segregation. Additionally, it was upregulated in pathways such as antigen processing and presentation of exogenous peptide antigen, as well as antigen processing and presentation of exogenous antigen. These enriched pathways are primarily associated with normal mitosis and cell division, providing evidence for the high proliferation status of this subgroup. This further underscores the importance of focusing on it as a key research subject.

We also examined the role of signaling pathways and related ligand-receptors pair in osteoclasts, analyzing interactions between osteoclasts and other cell types using Cellchat methods, particularly focusing on the crosstalk between C2 MKI67+ Osteoclast and tumor cells. Studies have shown that in outgoing signaling patterns, the APP signaling pathway was strongly expressed in some osteoclast subsets, while in incoming signaling patterns, the APP signaling pathway was mainly related to ECs and chondroblastic. Accordingly, we identified the significant signaling pathway APP and its related APP-CD74 ligand-receptor pair. The amyloid precursor protein (APP) pathway was a transmembrane precursor protein that was widely expressed in cell types such as osteoclasts, playing various roles in the human body. The APP pathway is also closely linked to the progression of various cancers, including lung cancer, pancreatic cancer, and colon cancer. CD74 is a type II transmembrane protein that acts as a receptor for APP and can be involved in regulating inflammatory and immune responses. In addition, studies have shown that CD74 can promote RANKL-induced osteoclast formation *in vitro* (82). In this study, the APP signaling pathway and its related APP-CD74 ligand-receptor pair in C2 MKI67+ Osteoclast played a positive role in osteoclast generation, inducing osteoclast activation, and enhancing osteoclast function (83–85).

In addition, we studied the metabolism of osteoclasts and found the most important metabolic pathway, oxidative phosphorylation, which was highly expressed in several osteoclast subsets. Oxidative phosphorylation (86–88) is the main process of energy production in cells. It is mainly the energy released by the oxidation step of organic matter (sugar, lipids, amino acids, etc.) in the decomposition process that drives the process of ATP synthesis. Oxidative phosphorylation (89) has a significant effect on osteoclasts, as osteoclasts have a high energy demand during the bone resorption process, and oxidative phosphorylation is the most efficient way for osteoclasts to obtain energy. Therefore, we hypothesize that the oxidative phosphorylation metabolic pathway could serve as a new therapeutic target. By targeting this metabolic pathway to inhibit osteoclast function, it may provide a strategy for treating OS (90).

Advances in single-cell technology had enabled further analysis of the high-dimensional transcriptomics of OS, identifying highly active tfs. TFs were proteins that could bind to specific DNA sequences to regulate gene expression (91). The most active TF in C2 MKI67+ Osteoclast was PPARG. Peroxisome proliferator-activated receptor (PPARG), a gene encoding the protein PPAR-γ, had been reported to increase fracture rates in patients when activated by agonists like rosiglitazone (92). This finding intrigued us. Research indicated that PPAR-γ had a strong pro-osteoclast function, and activating PPAR-γ could induce osteoclastogenesis and differentiation, maintain or increase bone resorption, and lead to bone homeostasis imbalance and destruction (93). The WNT/β-catenin pathway was usually expressed oppositely to PPARG, with PPAR-γ activation leading to WNT/β-catenin pathway downregulation, thereby inducing PGC1γ, promoting oxidative phosphorylation gene induction, and further inducing mitochondrial biogenesis essential for supporting osteoclast function (94).

This study presented the key osteoclast subpopulation and the target genes, signaling pathways, and TFs acting on this subpopulation. The target gene MKI67 promoted osteoclast proliferation and differentiation, the APP signaling pathway and its related the APP-CD74 ligand-receptor pair, and the transcription factor PPARG promoted osteoclast proliferation, differentiation, and bone resorption, playing significant roles in osteoclast function. Osteoclasts, in turn, contributed substantially to OS generation and progression. We speculated that PPARG and others might drive OS generation and progression by mediating osteoclasts, and targeting them to inhibit osteoclasts could indirectly affect OS generation and progression. In order to support the above speculation, we carried out *in vitro* experiments of PPARG. Through RT-qPCR detection, we found that knockdown of PPARG significantly inhibited osteoclast expression. In the Cell-Counting Kit-8 (CCK-8) Assay, we found that the proliferation ability of PPARG knockdown osteoclasts was significantly reduced, and the cell activity was significantly decreased. The wound healing assay and transwell assay showed that the migration ability of osteoclasts in the two groups of PPARG knockdown was weakened. Experiments have confirmed that PPARG has the ability to promote osteoclast proliferation and differentiation. The applicability of targeting PPARG has been confirmed, but its clinical application still needs to be continuously explored.

In summary, the target genes MKI67, APP signaling pathway (95), and its related receptors have the potential to become new therapeutic targets for APP-CD74 and transcription factor PPARG, which provides new ideas for OS treatment. The new targets proposed above help to promote the in-depth development of OS research and provide new strategies for the predictability, prevention, and personalized treatment of the disease. Although our content showed the heterogeneity of osteoclasts in OS and provided new insights into the treatment of OS, there were still some limitations. First of all, although many studies had shown that osteoclasts could promote OS, it had not been further verified in this paper. Secondly, we mainly relied on existing scRNA-seq data, which might not have been comprehensive and could have been biased. This paper focused solely on the analysis of single-cell data registered under GSE152048, which may introduce biases and impact the comprehensiveness and generalizability of the findings. Ultimately, we identified PPARG as a key factor through literature review and scRNA-seq, and its role in promoting osteoclast proliferation and differentiation was validated through cell experiments. However, as the experiments were conducted *in vitro*, the results could not fully confirm the migration and invasion capabilities of cells *in vivo*.

## Conclusion

Osteoclasts were an important part of OS. Studies have shown that osteoclasts could play a role in the progression and metastasis of OS. The C2MKI67+ osteoclast has been identified as a pivotal subgroup within the TME of OS. Experimental evidence highlighted specific targets, such as the transcription factor PPARG, as key regulators of osteoclast activity. The role of osteoclasts in OS revealed a promising therapeutic approach: strategically targeting osteoclasts to inhibit their proliferation and activity, thereby slowing the progression of osteosarcoma.

## Data Availability

The original contributions presented in the study are included in the article/**Supplementary Material**. Further inquiries can be directed to the corresponding authors.
