# Supplementary figures and images for: Single-cell RNA sequencing revealed PPARG promoted osteosarcoma progression: based on osteoclast proliferation
Source: Front Immunol. 2025 Jan 28;15:1506225. doi: 10.3389/fimmu.2024.1506225 (PMC11810940; doi:10.3389/fimmu.2024.1506225)

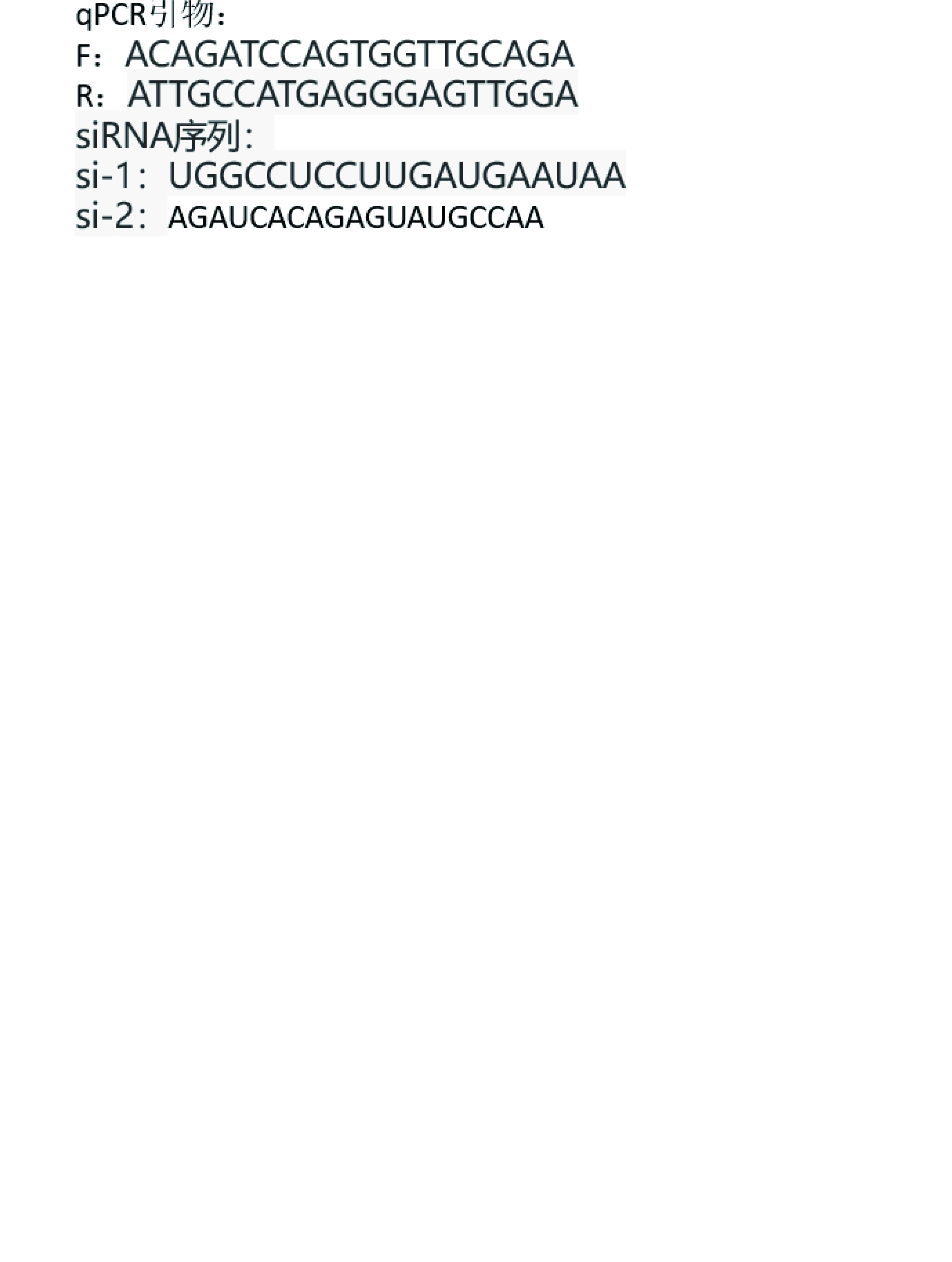

Supplement: Supplementary file 1 [file Image1.tif]
